# Research on real-time detection and staging technology for pressure injuries in critically ill patients based on the YOLOv8 deep learning model

**DOI:** 10.3389/fpubh.2026.1781481

**Published:** 2026-05-07

**Authors:** Fengbo Guo, Ningbo Cao, Jingyi Nie, Wenwen Guo, Juzi Wang

**Affiliations:** 1 Shanxi Provincial People's Hospital, Taiyuan, Shanxi, China; 2 The nursing Department of Shanxi Provincial People's Hospital, Taiyuan, Shanxi, China

**Keywords:** deep learning, image processing, object detection, pressure injury, YOLO

## Abstract

**Background:**

Aiming to address the clinical challenges of high incidence rates of pressure injuries (PIs) in critically ill patients and the subjectivity and inefficiency of traditional assessment methods, this study developed a real-time detection and staging technology based on the YOLOv8 deep learning model. This technology enables rapid and objective staging of PIs, aiming to overcome the limitations of current manual assessments and provide support for precision nursing care.

**Objective:**

To develop and validate a real-time detection and staging system for PIs using the YOLOv8 deep learning model.

**Study design:**

A total of 507 PI images from intensive care unit patients (Jan 2023–Jun 2025) were randomly divided into training (414) and test (93) sets at an 8:2 ratio. Images were classified into six stages per NPUAP guidelines. Five YOLOv8 versions were developed using transfer learning, with AdamW optimizer and dynamic learning rate adjustment. The best model was evaluated on accuracy, mean average precision (mAP), and inference speed.

**Results:**

This model effectively enhanced the objectivity and accuracy of pressure injury (PI) staging identification. In testing with 93 PI images, YOLOv8l achieved an overall accuracy of 96.8% and a mAP@50 of 0.847, while maintaining an inference speed of 28.6 FPS (frames per second)—outperforming other versions. Additionally, the model demonstrated high prediction accuracy across all six PI stages: all Stage 2, Stage 4, and Unstageable images were correctly predicted; one image each in Stage 1, Stage 3, and Deep Tissue Injury (DTI) was misclassified.

**Conclusions:**

For PI staging identification, the PI assessment system built on the YOLOv8l deep learning model demonstrates high accuracy and efficiency, providing reliable support for clinical decision-making.

**Relevance to clinical practice:**

This system enables objective PI evaluation and personalized care planning, which may reduce PI-related complications and associated healthcare costs.

## 1 Introduction

Pressure injury (PI), also known as pressure ulcer (1), refers to localized tissue damage caused by intense and/or prolonged pressure, or the combined effects of pressure and shear force, affecting the skin and underlying soft tissues. These injuries typically occur over bony prominences, presenting clinically as damage beneath intact skin or open ulcers. They may also involve mucosal tissues (such as the mucosal surface, submucosa, or submucosal layer) or be associated with medical device use. The injury may be painful or painless, and its development is influenced by multiple factors including the microenvironment, nutritional status, and complications. The intensive care unit (ICU) is a specialized setting for the treatment of critically ill patients. Due to the severity of their conditions, ICU patients often require multiple supportive medical devices, prolonged bed rest, and frequently exhibit sensory impairment, reduced communication and mobility abilities, and altered consciousness. These factors can lead to prolonged pressure on the skin and subcutaneous tissues, ultimately resulting in PI (2). Research statistics indicate that compared to the 5%−15% PI incidence rate among general inpatients (3), the rate reaches 15%−25% during ICU stays. This increases patients' susceptibility to infections, prolongs hospital stays, and incurs treatment costs that exceed expenditures allocated by individuals and society for health prevention through medical insurance. As frontline caregivers for critically ill patients, ICU nurses play a crucial role in reducing PI incidence through accurate assessment and appropriate nursing interventions (4). However, traditional assessment methods are influenced by multiple factors, including individual knowledge gaps, inadequate training, subjective judgment, and environmental variables like lighting and skin discoloration (5). These limitations not only increase workload but may also cause patient discomfort during measurement, with varying reliability of outcomes (6). Precise diagnosis and assessment of PI enable healthcare providers to objectively evaluate PI staging and select treatment modalities, thereby delivering more personalized care to critically ill patients and significantly reducing PI-related healthcare costs (7).

In recent years, the continuous advancement of artificial intelligence through deep learning has provided favorable conditions for researchers in the field of intelligent healthcare (8). Convolutional neural networks (CNNs), as a machine learning algorithm within deep learning, are widely employed for tasks like wound segmentation and classification due to their efficient image processing capabilities (9, 10). Faster region-based convolutional neural networks (R-CNNs) demonstrate greater accuracy in detecting wound boundaries and performing image detection and classification, though they demand higher computational resources and longer training times (11, 12). In contrast, YOLO (You Only Look Once), introduced in 2015 and refined through multiple iterations, has emerged as a cutting-edge real-time object detection AI solution (13). Unlike the two-stage detection strategy (candidate region generation + precise localization) of traditional models like R-CNN, YOLO transforms object detection into a regression problem, completing detection with a single image scan. This approach achieves faster execution and reduced computational resource requirements (14). The 2023 YOLOv8 introduced an anchor-free mode, enabling direct prediction of object center, size, and shape without predefined anchor boxes (15). This approach streamlines the detection process, making it faster and more efficient, particularly for real-time medical image analysis (16). However, domestic research on utilizing deep learning for automated PI staging remains scarce. Therefore, addressing the current lack of precise and unified standards for clinical nurses' subjective assessment of PI staging features, this study develops a novel technology for real-time detection and staging of PIs based on the YOLOv8 neural network model. It aims to provide clinicians with a reliable PI staging tool, improve patient treatment outcomes, and alleviate the burden on healthcare professionals.

## 2 Materials and methods

### 2.1 Study population

This study selected 1,024 images of PI from patients admitted to our hospital's intensive care unit between January 2023 and June 2025. After screening, 507 images met the inclusion criteria for analysis. All data were stored in the hospital's dedicated electronic PI information management system.

### 2.2 Data collection

Nursing staff capture patients' PI images in clinical settings using medical PDAs or personal mobile phones under natural lighting. Images are taken at a distance of 50 cm while maintaining stability during capture. Completed images are subsequently uploaded to the electronic PI information management system.

### 2.3 Image annotation

**Figure 1:**
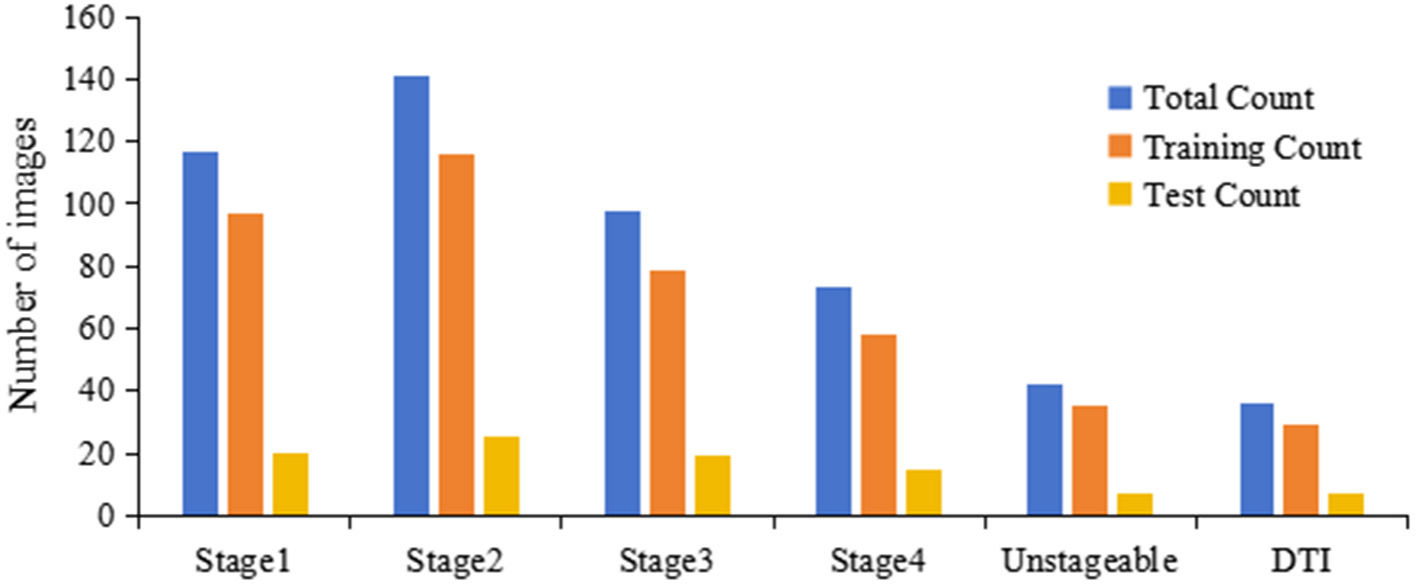
The number of images across different pressure injuries stages.

**Figure 2:**
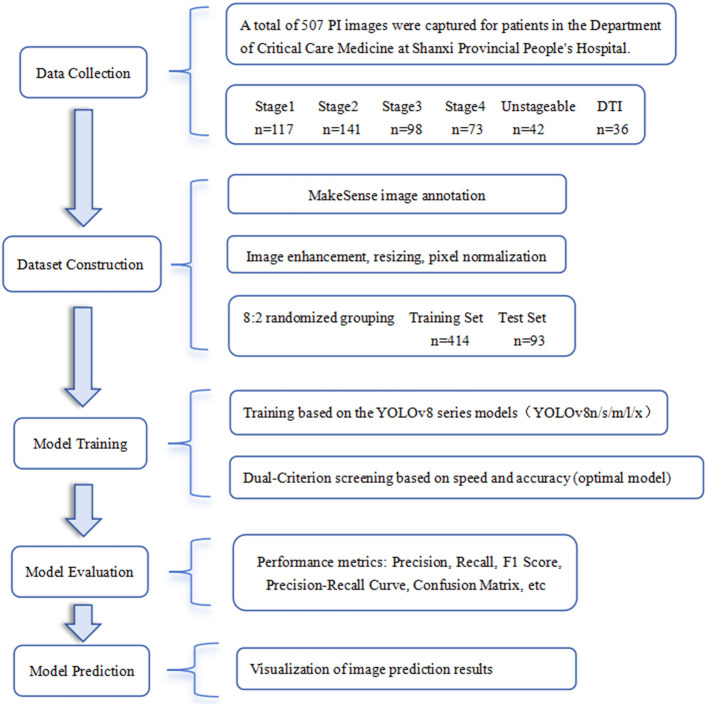
Research flowchart.

This study obtained a total of 1,024 images. After excluding images with poor resolution, overexposure or underexposure, inadequate lighting, and cluttered backgrounds, 507 high-resolution images with appropriate focal length, sufficient lighting, and minimal noise were selected. These were randomly divided into a training set (414 images) and a test set (93 images) at an 8:2 ratio. Image annotation was performed collaboratively by three experienced Wound, Ostomy, and Continence (WOC) nurses (with 8, 12, and 15 years of clinical experience, respectively) from the hospital. Following the 2019 Pressure Ulcer Prevention and Treatment Guidelines published by the National Pressure Ulcer Advisory Panel (NPUAP) (17), images were classified into six stages. Prior to consensus building, inter-rater reliability was assessed using Fleiss' Kappa, which yielded a value of 0.73 (95% CI: 0.68–0.78), indicating substantial agreement. Disagreements were resolved through discussion to establish the final reference standard. Image annotation was performed using the Make Sense online annotation tool, with annotation files exported in YOLO data format. These verified annotated images were subsequently used to train the deep learning model. Reference criteria for each stage are shown in Table 1 (18), the distribution of images across stages is illustrated in Figure 1, and the study workflow is depicted in Figure 2.

**Table 1:** Staging of pressure injuries.

Staging	Definition
Stage 1	Intact skin with localized non-blanchable erythema; presentation may differ in darker skin tones. Erythema that blanches to pressure, or changes in sensation, temperature, or firmness, may precede visible skin changes.
Stage 2	Partial-thickness skin loss with dermal exposure. The wound bed is active, pink or red, and moist, or may present as intact or ruptured serous blisters. No subcutaneous fat or deep tissue exposure; no granulation tissue, slough, or eschar.
Stage 3	Full-thickness skin loss with exposed subcutaneous fat. Granulation tissue or epibole are commonly present, with possible local necrotic tissue and/or eschar. Areas rich in adipose tissue may exhibit deeper defects. Undermining and tunneling may develop, but fascia, muscle, tendon, ligament, cartilage, and/or bone exposure is absent.
Stage 4	Ulceration with full-thickness skin and tissue loss, accompanied by visible or palpable exposure of fascia, muscle, tendon, ligament, cartilage, or bone. Necrotic tissue and/or eschar may also be present locally. Typically associated with epibole, undermining and/or tunneling.
Unstageable	Although full-thickness skin and tissue loss is present, the extent of the defect is difficult to determine due to local necrotic tissue and/or eschar coverage. Removal of necrotic tissue and/or eschar would confirm a Stage 3 or Stage 4 pressure injury.
Deep tissue injury (DTI)	Skin may be intact or intact with persistent deep red, maroon, or purple discoloration that does not blanch with pressure. Alternatively, separation of the epidermis may reveal a black eschar or a hyperemic blister. Pain and temperature changes often precede skin discoloration. Color changes in darker skin tones may vary.

### 2.4 Preprocessing

To enhance the model's detection accuracy and generalization capability, preprocessing and augmentation operations were applied to the image data. Various modifications were implemented, including random vertical and horizontal flipping, rotation between 0 and 90 degrees, and brightness reduction. Furthermore, the images were resized to 224 × 224 pixels, and their pixel intensity values were normalized to the range [0, 1] to improve training stability.

### 2.5 Training configuration

This study employed a transfer learning strategy (19), utilizing five versions of the YOLOv8 model pre-trained on the large-scale COCO dataset: YOLOv8n, YOLOv8s, YOLOv8m, YOLOv8l, and YOLOv8x. These models differ in parameter count and inference speed, making them suitable for different scenarios. We retained the shallow layers of the pre-trained models and fine-tuned the deeper layers. During training, the AdamW optimizer was selected, and the learning rate was adjusted according to the configuration file. The learning rate was halved every 5 training epochs to stabilize training and achieve fine-tuning. The training was set for 100 epochs, incorporating the Early Stopping technique to terminate training early if no improvement was observed. The batch size was set to 20 and adjusted based on GPU memory availability to ensure stable training. Additionally, YOLO8's default compound loss function was used to balance localization and classification losses, and automated mixed-precision training on the GPU was enabled to increase training speed and reduce memory usage.

### 2.6 Evaluation metrics

In this study, we employed several metrics to evaluate model performance, including precision, recall, F1-score, mAP@50, and mAP@50–95. Precision (Equation 1) refers to the proportion of samples predicted as positive by the model that are actually true positives. Recall (Equation 2) refers to the proportion of actual true positive samples that are correctly predicted as true positives by the model, measuring the model's completeness. The F1-score (Equation 3) is the harmonic mean of precision and recall, providing a comprehensive evaluation of the model's performance.


$$\begin{array}{l} \text{Precision}=\;\frac{True\;Positives}{True\;Positives\;+\;False\;Positives} \end{array}$$ (1)

$$\begin{array}{l} Recall\;=\;\frac{True\;Positives}{True\;Positives+False\;Negatives} \end{array}$$ (2)

$$\begin{array}{l} F1-score\;=\;\frac{2\;\times \;Precision\;\times \;Recall}{Precison\;+\;Recall} \end{array}$$ (3)

mAP is a common metric for evaluating models, measuring the overall performance across different categories. mAP@50 refers to the mean average precision when the IoU threshold is set to 0.5, while mAP@50–95 represents the average precision of the model across IoU thresholds ranging from 0.5 to 0.95. Additionally, the confusion matrix was used to visualize prediction results across various categories, aiding in the analysis of the model's classification effectiveness for different stages. Furthermore, evaluation based on frame rate was conducted to reflect the system's real-time image processing capability, ensuring the requirement for immediate feedback in clinical applications.

### 2.7 Experimental platform

This study was conducted on a high-performance computing platform equipped with an NVIDIA RTX 3,080 Ti GPU (12GB VRAM), an Intel Xeon Platinum 8,369B processor, and 1TB of storage. Model development, training, and optimization were performed using the PyTorch 2.0 deep learning framework (with CUDA 11.8 acceleration library). Data processing and statistical analysis primarily relied on the Pandas 1.3.5 and NumPy 1.21.3 libraries. Result visualization tasks were implemented using Seaborn 0.12.2 and Plotly 5.12.0.

## 3 Results

### 3.1 Model training

**Figure 3:**
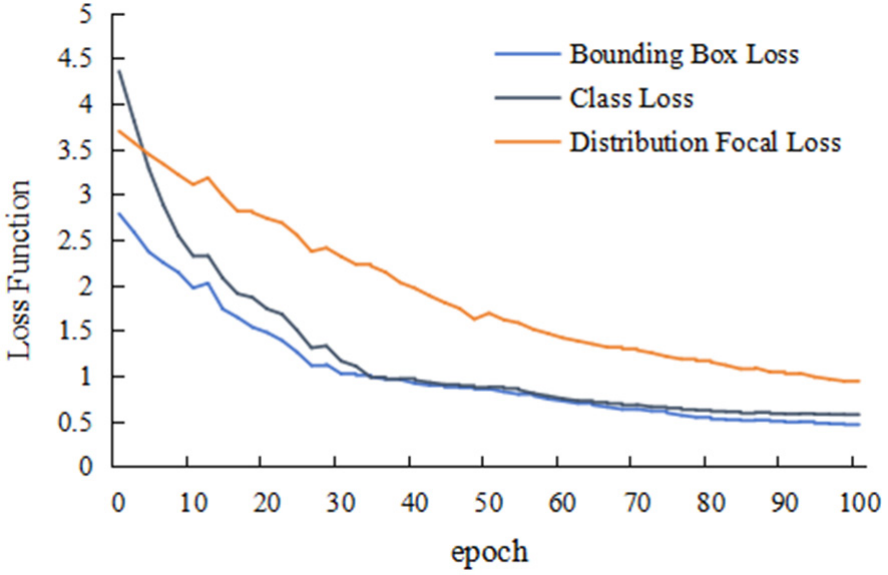
Loss function trends during YOLOv81 model training.

**Figure 4:**
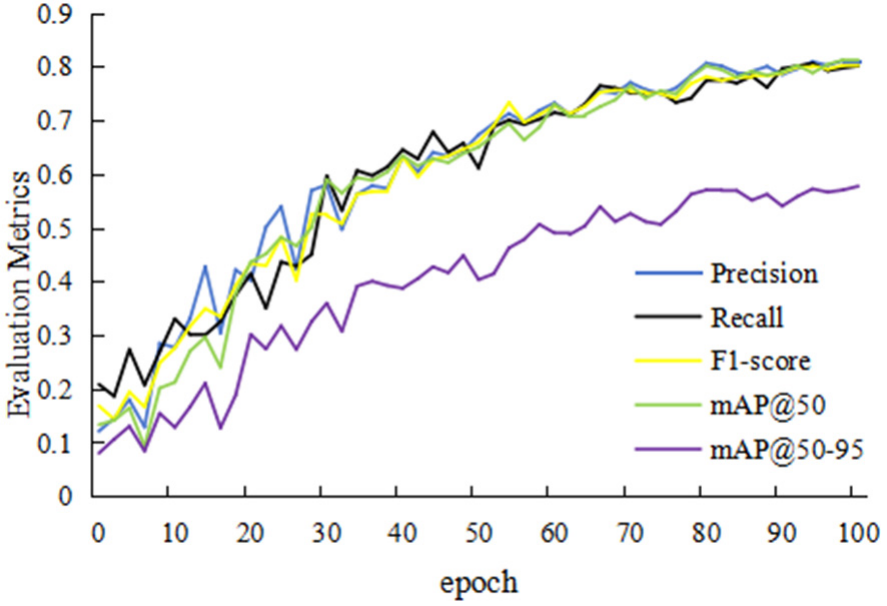
Trends of evaluation metrics during YOL Ov81 model training.

Figure 3 illustrates the evolution of loss functions during YOLOv8 training. Bounding box loss represents the discrepancy between the coordinates of the predicted and ground truth bounding boxes. Class loss indicates the probability of each detected object belonging to a specific category. The Distribution Focal Loss, built upon focal loss, enhances the model by distributing the focal loss across multiple scales and classes. The figure demonstrates a declining trend in all loss values as training progresses, indicating that the model is converging toward optimization. Concurrently, Figure 4 shows an increasing trend in the model's precision, recall, and mAP with the progression of training epochs. This suggests that the model's capability to detect PI at different stages in images continuously improves as the number of training iterations increases.

### 3.2 Model performance evaluation

#### 3.2.1 Bounding box regression prediction performance of different YOLOv8 model versions

Based on Table 2, the results show that the detection performance of the YOLOv8 series models generally improves with increasing complexity. Among them, YOLOv8l achieves an mAP@50 of 0.847, significantly outperforming YOLOv8n (0.762), YOLOv8s (0.788), YOLOv8m (0.796), and YOLOv8x (0.820), demonstrating the best detection metrics. In terms of classification accuracy, YOLOv8l leads the other versions with 96.8%, an increase of 8.6 percentage points compared to YOLOv8n's 88.2%. Notably, although YOLOv8x has a larger scale, its accuracy slightly decreases, suggesting that YOLOv8l may have achieved a better balance between model complexity and generalization performance. Additionally, processing speed decreases as model complexity increases, from 167 FPS for YOLOv8n to 28.6 FPS for YOLOv8l, while still meeting clinical real-time requirements. In summary, YOLOv8l achieves an optimal balance among detection performance, inference efficiency, and generalization performance, making it the most reliable model in this study. Detailed comparisons are presented in Table 2.

**Table 2:** Performance comparison of YOLOv8 variants on the PI staging test set.

Model version	Precision	Recall	F1-Score	mAP@50	mAP@50–95	Accuracy (%)	Speed (FPS)
YOLOv8n	0.741	0.757	0.749	0.762	0.532	88.2	167
YOLOv8s	0.786	0.728	0.756	0.788	0.546	91.4	100
YOLOv8m	0.802	0.772	0.787	0.796	0.552	93.5	56
YOLOv8l	0.854	0.746	0.796	0.847	0.577	96.8	28.6
YOLOv8x	0.827	0.737	0.779	0.820	0.560	95.3	25

#### 3.2.2 Performance of the YOLOv8l model at different confidence thresholds

**Figure 5:**
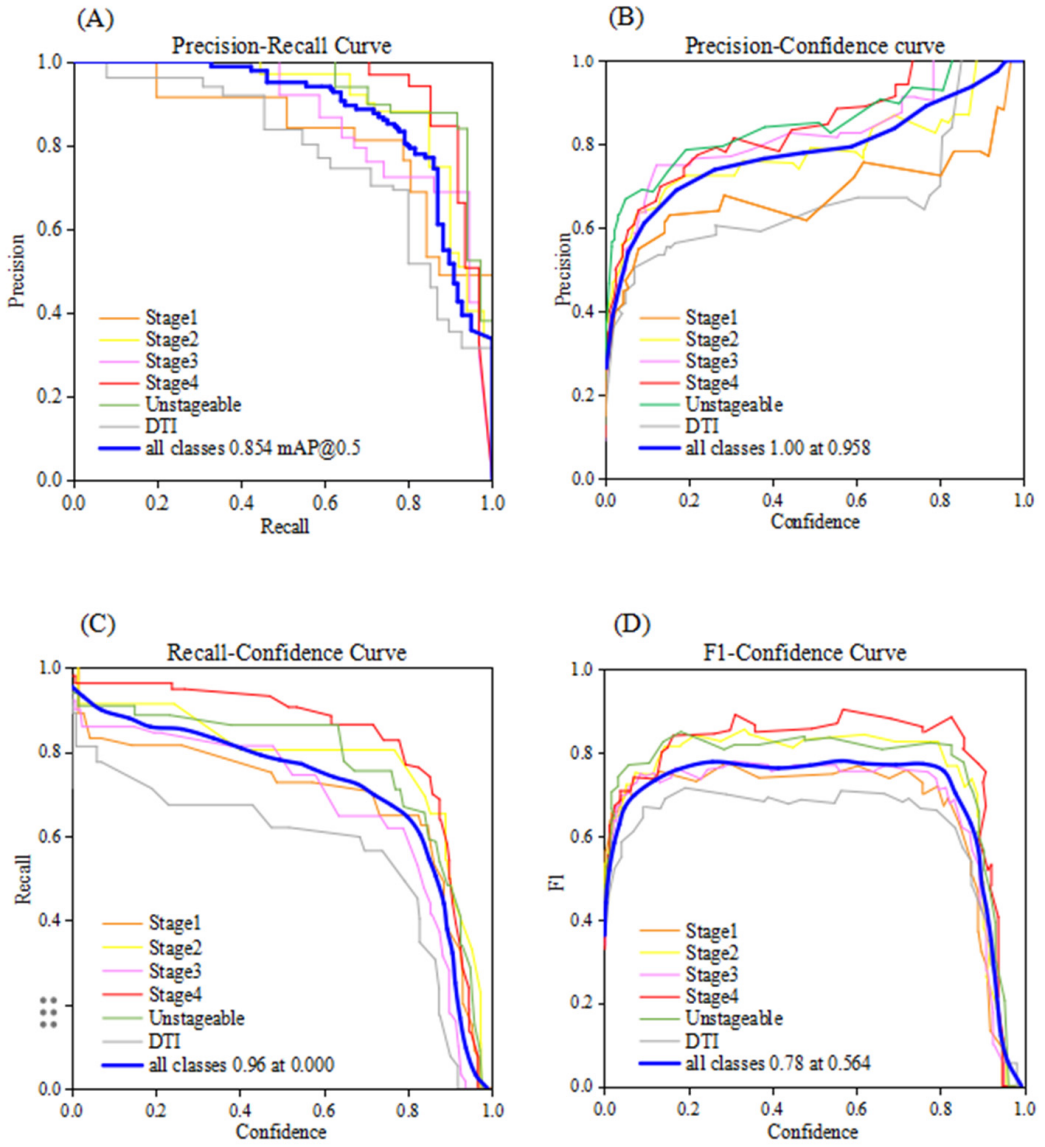
Composite performance curves of the YOLOv81 model: **(A)**. Precision –recall curve, **(B)**. Precision-confidence curve, **(C)**. Recall-confidence curve, **(D)**. F1-confidence curve.

As shown in Figure 5, the model's performance across different confidence thresholds was systematically evaluated using the Precision-Recall curve, Precision-Confidence curve, Recall-Confidence curve, and F1 Score-Confidence curve. The P-R curve illustrates the dynamic trade-off between precision and recall, providing critical guidance for model threshold optimization. The Precision-Confidence and Recall-Confidence curves depict the trends of precision and recall at varying confidence levels, respectively. They indicate that the model effectively enhances lesion screening sensitivity at lower thresholds while significantly improving detection specificity at higher thresholds. In the F1-Confidence curve, the F1 score initially increases and then decreases with rising confidence, reaching a peak value of 0.78 for all classes at a confidence of 0.564, achieving the optimal balance between precision and recall. Combined with subsequent validation by the PI nursing team, this multi-dimensional confidence analysis establishes the model's reliability and practicality in real-world applications. Overall, the results from the YOLOv8l model demonstrate its accurate detection and classification of PIs within the training and validation datasets.

#### 3.2.3 Predictive performance of the YOLOv8l model for PI staging

**Figure 6:**
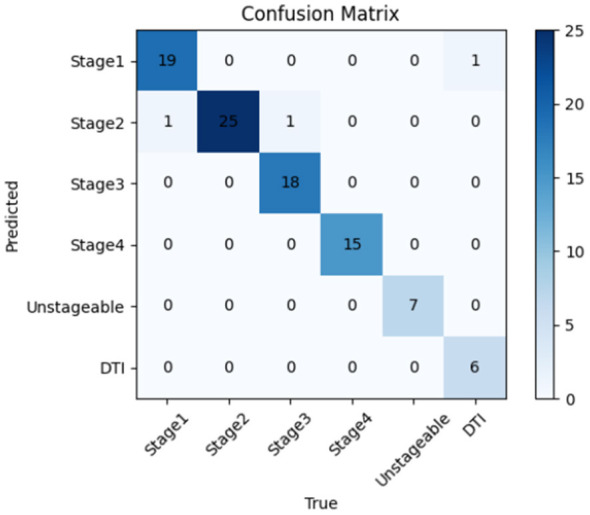
Confusion matrix of the YOLOv81 model on the PI staging dataset.

**Figure 7:**
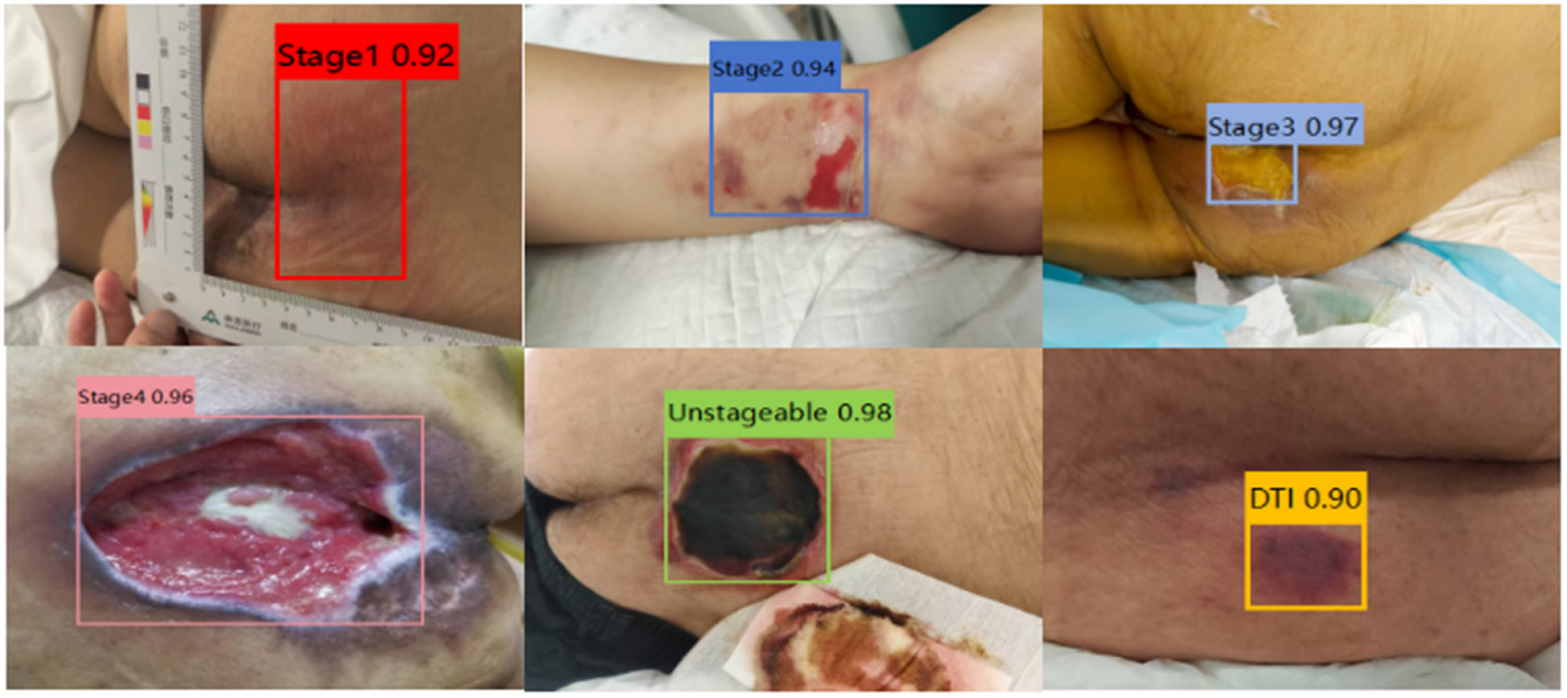
Representative test results of the YOLOv18 model for PI staging.

The YOLOv8l model was used to predict 93 PI images at different stages from the test set, and a confusion matrix was constructed by comparing the predictions with the ground truth labels (Figure 6). As shown in the figure, all Stage 2, Stage 4, and Unstageable images were correctly predicted, achieving 100% accuracy. However, one image each from Stage 1, Stage 3, and DTI was misclassified. This indicates that the network model performs well in identifying PI stages, particularly for Stage 2, Stage 4, and Unstageable images. To further illustrate the nature of these misclassifications, Table 3 summarizes the three erroneous cases along with their possible causes. Based on the confusion matrix of the test set, the sensitivity and specificity of the YOLOv8l model for each stage were calculated (Table 4). The specificity for all stages was ≥97.1%, indicating an extremely low misdiagnosis rate. Additionally, Figure 7 displays representative image analysis results detected by the YOLOv8l model, including the stage, confidence score, and visual annotations. The visualization of prediction outcomes enhances their credibility.

**Table 3:** Summary of misclassified cases in the test set.

True stage	Predicted stage	Count	Possible cause
Stage 1	Stage 2	1	Localized erythema with possible early superficial disruption, potentially misinterpreted as partial-thickness loss
Stage 3	Stage 2	1	Full-thickness wound with unclear depth boundaries due to lighting conditions or granulation tissue obscuring the defect
DTI	Stage 1	1	Subtle dark discoloration of DTI may resemble persistent erythema of Stage 1 under certain lighting conditions

**Table 4:** Sensitivity and specificity of the YOLOv8l model in each stage of PI.

Staging	Sensitivity	Specificity	Sample size for test
Stage 1	0.950	0.986	20
Stage 2	1.000	0.971	25
Stage 3	0.947	1.000	19
Stage 4	1.000	1.000	15
Unstageable	1.000	1.000	7
DTI	0.857	1.000	7

## 4 Discussion

PI is a common and serious complication in patients with prolonged bed rest or limited mobility, such as critically ill patients, and its incidence is closely associated with mortality. The Global Burden of Disease study report indicates that China has become a high-incidence country for PI due to the dual effects of an accelerating aging population and imbalanced allocation of medical resources (20). PI not only causes severe pain and increases the risk of infection in patients, but the prolonged and difficult healing process also significantly extends hospital stays and consumes medical resources (21). Clinical prevention and management face multiple challenges. Traditional assessment relies on manual observation, which is subjective, time-consuming, and labor-intensive, making it difficult to detect early-stage damage promptly, especially in intensive care settings (22). Staging diagnosis requires extensive professional expertise, and delays in judgment can easily lead to deterioration of the condition. Although multidisciplinary collaboration and technologies such as advanced dressings are continuously advancing, there remains an urgent need for real-time, accurate, and automated detection and staging methods. Lei et al. investigated the capability of four different CNN models for classifying PI images (23). Their results showed that DenseNet121 achieved the highest classification accuracy of 93.71%, demonstrating strong performance in PI image classification. Ge et al. compared a CNN-based AI system with traditional assessment methods for PI evaluation (24). The results indicated that the AI system achieved an accuracy of 90%, superior to the 81.2% recognition accuracy of traditional methods, significantly reducing assessment time and improving the overall efficiency of PI evaluation. Chen et al. established an intelligent telemedicine diagnosis system based on YOLOv7 and a large language model to assist users in staging and classifying PI, providing real-time and accurate diagnostic and treatment suggestions (25). In contrast, this study developed a real-time detection and staging model for PIs in critically ill patients based on the YOLOv8 neural network. Validation on a clinical dataset demonstrated its capability for rapid identification and accurate staging of PI. This holds urgent practical significance and clinical value for reducing medical costs and improving patient outcomes, representing an important exploration direction for intelligent nursing and precision medicine. The following discussion centers on the research findings, limitations, and future directions.

### 4.1 Research findings

This study achieved significant results by applying the YOLOv8l model for PI detection. The model demonstrated exceptional performance with an overall accuracy of 96.8%, particularly excelling in the detection of Stage 2, Stage 4, and Unstageable PIs. This validates its efficient object detection capability and the precise capture of injury characteristics through multi-scale feature fusion technology. The model exhibited outstanding real-time performance (processing speed: 28.6 FPS), which corresponds to an inference time of approximately 0.035 s per image. This means the model can analyze nearly 29 images per second, enabling near-instantaneous assessment upon image capture and fully meeting the stringent requirements for rapid response in critical care scenarios. Although minor errors occurred in Stage 1, Stage 3, and DTI, considering that it might be due to lighting issues and staging complexity, the model successfully identified a high proportion of PIs overall. The Stage 1 image misclassified as Stage 2 exhibited localized erythema with possible superficial disruption, potentially misinterpreted as partial-thickness loss. The Stage 3 image misclassified as Stage 2 likely involved unclear depth boundaries due to lighting or granulation tissue obscuring the full-thickness defect. The DTI misclassified as Stage 1 is particularly noteworthy, as DTI can present with subtle dark discoloration resembling Stage 1 erythema under certain lighting. A recent study reported that the most common staging disagreements occur precisely between DTI and Stage 1, as well as between DTI and Stage 2 (26). These observations highlight that visual feature overlap and image acquisition conditions can impact model judgment, underscoring the need for standardized image capture protocols and targeted data augmentation. Further analysis of sensitivity and specificity showed that the model achieved 100% sensitivity for Stage 2, Stage 4, and Unstageable. Sensitivity was 95.0% for Stage 1, 94.7% for Stage 3, and 85.7% for DTI. The high detection rates for Stage 4 and Unstageable—which may be partially attributed to their relatively distinctive visual features (exposed bone in Stage 4; full-thickness tissue loss covered by slough/eschar in Unstageable)—are similar to those reported in a recent meta-analysis demonstrating high diagnostic accuracy for these stages (AUC: 0.94 for Stage 4, 0.96 for Unstageable) (27). However, the small sample sizes for Unstageable (*n* = 7) and DTI (*n* = 7) warrant cautious interpretation and validation in larger cohorts. The consistently high specificity (≥97.1%) across all stages indicates an extremely low misdiagnosis rate, underscoring the model's clinical safety and reliability. Compared to traditional manual assessment methods, the technical advantages of YOLOv8l lie in its real-time automated processing, objective and standardized staging, and scalability, effectively avoiding subjective variability and providing a critical time window for early warning. Its clinical value is reflected in: optimizing personalized care decisions (such as pressure relief measures and debridement timing) through accurate staging results, thereby reducing the risk of injury progression; simultaneously, it alleviates healthcare professionals from repetitive tasks, enabling a greater focus on complex clinical judgments. The model's scalability gives it the potential for integration into existing monitoring systems, laying the foundation for intelligent and precise development in the ICU. This represents a deep integration of technological innovation and clinical practice, providing an efficient and reliable AI solution for PI management.

### 4.2 Study limitations

1. Dataset bias: The dataset used was not adequately large in sample size, the distribution ratio of samples across different stages was uneven, and the samples were solely sourced from a single center. The model has not undergone independent testing with external datasets and lacks generalization verification with multi-center data. This may limit its applicability in other populations, medical environments, or under different lighting conditions.
2. Identification of DTI: The detection accuracy for complex samples requires improvement. Moreover, since changes in tissue pain and temperature often precede skin color changes, future work could incorporate synthetic data augmentation or integrate multi-modal information (e.g., infrared thermal imaging) to enhance performance.
3. Depth of clinical validation: The current study only validated the model's technical performance. Prospective controlled clinical trials have not yet been conducted to assess its actual impact on patient outcomes.
4. Integrated platform development: Although this study has completed the research on the functional components of a high-performance PI image processing and recognition system, a fully integrated and deployable central detection platform has not been realized.

### 4.3 Future work

1. Carry out multi-center external validation, collaborate with multiple medical institutions, and construct a diversified dataset of pressure injuries covering different regions, populations, and equipment conditions. Conduct independent external tests on the model to comprehensively evaluate its generalization ability and robustness.
2. Multi-modal fusion: Improve comprehensive decision-making by integrating clinical data (e.g., pressure and temperature sensors, vital signs) with image information.
3. Conduct randomized controlled trials: Quantify the model's effect on improving nursing efficiency and patient outcomes through rigorous randomized controlled trials (RCTs).
4. Edge computing optimization: Further compress the model parameters to enable real-time inference on low-power devices, meeting the requirements for bedside monitoring.

### 4.7 Personalized intervention strategies

Develop an AI-driven recommendation system for personalized nursing care plans based on injury characteristics and staging results.

## 5 Conclusion

This study developed a methodological framework for real-time detection and staging of PIs in critically ill patients based on YOLOv8l, validating its technical feasibility and potential value in clinical scenarios. The model's high efficiency and accuracy provide a powerful new tool for the early detection and intervention of PIs, promising to become an important means of supporting clinical decision-making in wound assessment and helping to reduce the risk of complications and the healthcare burden for critically ill patients. With the future enrichment of PI image databases and advances in cutting-edge technologies, multi-center validation, technical optimization, and clinical empirical studies will continue to promote the in-depth application of AI in critical care nursing.

## Data Availability

The raw data supporting the conclusions of this article will be made available by the authors, without undue reservation.
